# Discovery of gorilla MHC-C expressing C1 ligand for KIR

**DOI:** 10.1007/s00251-017-1038-y

**Published:** 2017-11-03

**Authors:** Jörg B. Hans, Linda Vigilant

**Affiliations:** Max Planck Institute for Evolutionary Anthropology, Deutscher Platz 6, 04103 Leipzig, Germany

**Keywords:** MHC genotyping, PacBio, Next-generation sequencing, Bottleneck, Evolution

## Abstract

In comparison to humans and chimpanzees, gorillas show low diversity at MHC class I genes (*Gogo*), as reflected by an overall reduced level of allelic variation as well as the absence of a functionally important sequence motif that interacts with killer cell immunoglobulin-like receptors (KIR). Here, we use recently generated large-scale genomic sequence data for a reassessment of allelic diversity at *Gogo-C*, the gorilla orthologue of *HLA-C*. Through the combination of long-range amplifications and long-read sequencing technology, we obtained, among the 35 gorillas reanalyzed, three novel full-length genomic sequences including a coding region sequence that has not been previously described. The newly identified Gogo-C*03:01 allele has a divergent recombinant structure that sets it apart from other *Gogo-C* alleles. Domain-by-domain phylogenetic analysis shows that Gogo-C*03:01 has segments in common with Gogo-B*07, the additional *B*-like gene that is present on some gorilla MHC haplotypes. Identified in ~ 50% of the gorillas analyzed, the Gogo-C*03:01 allele exclusively encodes the C1 epitope among Gogo-C allotypes, indicating its important function in controlling natural killer cell (NK cell) responses via KIR. We further explored the hypothesis whether gorillas experienced a selective sweep which may have resulted in a general reduction of the gorilla MHC class I repertoire. Our results provide little support for a selective sweep but rather suggest that the overall low *Gogo* class I diversity can be best explained by drastic demographic changes gorillas experienced in the ancient and recent past.

**Electronic supplementary material:**

The online version of this article (10.1007/s00251-017-1038-y) contains supplementary material, which is available to authorized users.

## Introduction

Molecules encoded by the MHC class I genes are an integral component of the adaptive and innate immune system. Their primary function is to bind antigens of intracellular origin and present them on the cell surface to immunocompetent cells. Upon recognition of pathogen-derived antigens, cytotoxic CD8+ T cells are activated to initiate an appropriate immune response, ultimately leading to the lysis of the infected cell (Davis and Bjorkman 1988; Wong and Pamer 2003). Some MHC class I allotypes also interact with NK cells via KIR. Cells showing reduced or aberrant expression of MHC class I molecules due to viral infection or malignant transformation are eliminated by NK cells whose cytolytic activity is regulated by activating and inhibitory KIR (Vilches and Parham 2002; Lanier 2005).

During primate evolution, the MHC class I multigene family has recurrently expanded and contracted in structure and gene number (Kulski et al. 2002; Kelley et al. 2005; Bontrop 2006). As a result of the “birth-and-death” evolutionary process, the MHC class I multigene family consists of a variety of genes: highly polymorphic genes encoding classical MHC class I molecules, conserved genes encoding nonclassical MHC class I molecules, and various pseudogenes and gene fragments (Piontkivska and Nei 2003; Nei and Rooney 2005). Further, the varied coevolutionary dynamics between pathogens and their hosts have shaped the genes encoding the MHC class I molecules leading to species-specific patterns of intra- and interlocus variability (Hughes and Nei 1989; Adams and Parham 2001).

Indeed, orthologues of all classical human MHC class I genes (*HLA-A*, *-B*, and *-C*) can be found in the MHC of the gorilla (*Gogo-A*, *-B*, and *-C*), but not vice versa. Distinguishing the gorilla MHC is a polymorphic *A*-related locus present on some haplotypes, designated *Gogo-Oko*, which has no functional human orthologue (Lawlor et al. 1991; Watkins et al. 1991; Gleimer et al. 2011; Hans et al. 2017). Furthermore, there is evidence for differences in the copy number of certain *Gogo* class I genes. Whereas all human haplotypes have a single copy of the classical *HLA* class I genes, some gorilla MHC haplotypes are characterized by an additional *B*-like gene (Hans et al. 2017). However, despite gorillas having a comparatively complex haplotype structure, their MHC class I diversity appears to be low, which is reflected not only by an overall reduced level of allelic variation but also by the absence of a functionally important sequence motif at *Gogo-C*, the gorilla orthologue of *HLA-C* (Hans et al. 2017). Specifically, all previously described Gogo-C allotypes are predicted to express only one of two KIR-binding epitopes, the C2 epitope, whereas the C1 epitope is carried only by a minority of Gogo-B allotypes (Lawlor et al. 1991; Urvater et al. 2001; Adams and Parham 2001; Martínez-Laso et al. 2006; Hans et al. 2017). In contrast, MHC-C allotypes of humans and chimpanzees carry either the C1 or the C2 epitope, which suggests that gorillas have lost the C1-bearing allotypes at this locus (Moesta et al. 2009; Parham et al. 2012; Hans et al. 2017).

Here, we extend our previous work characterizing MHC class I genes in gorillas. By using recently generated long-read genomic sequence data, we provide a reassessment of the *Gogo-C* gene variation, revealing that gorillas possess a single *Gogo-C* allele that expresses the C1 ligand for KIR. In addition, we extend analyses exploring the hypothesis whether gorillas experienced a selective sweep which might have resulted in the reduction of their MHC class I repertoire.

## Material and methods

### Virtual MHC genotyping and primer design

Recently, a high-quality genome assembly of the female western lowland gorilla (*Gorilla gorilla gorilla*) named “Susie” was generated using long-read sequencing technology (Gordon et al. 2016). To investigate the *Gogo* class I alleles of “Susie,” we downloaded from the European Nucleotide Archive (ENA) the contig (000730_F) spanning the MHC class I region. Comparison to our previously reported full-length *Gogo* class I alleles (Hans et al. 2017) showed that this haplotype contains the following alleles: Gogo-A*01:01:01, Gogo-A*05:02:01:02N, and Gogo-B*01:01:01:01. However, in the expected genomic position of *Gogo-C* located approximately 85 kb upstream of *Gogo-B*, the contig showed only partial sequence similarity to known *Gogo-C* alleles. Virtual genotyping with the *Gogo-C* primers used in our previous study revealed that the contig sequence contains a single base pair insertion causing mismatches at the 3′-end of the reverse primer located in the 3′ untranslated region (3’UTR) (Online Resource 1). Although it must be noted that PacBio sequencing errors predominantly consist of single insertions and deletions, these findings strongly suggest that we may have underestimated allelic variation at the *Gogo-C* gene in our previous study due to the non-amplification of certain alleles (Ross et al. 2013; Laehnemann et al. 2016; Hans et al. 2017).

To assess this possibility, we designed a new primer pair for the long-range PCR (LR-PCR) amplification of *Gogo-C* to re-evaluate allelic diversity at this locus. From the abovementioned contig, we extracted the sequence comprising the putative *Gogo-C* gene and generated a ClustalW multiple alignment together with all available MHC class I gene sequences from human, chimpanzee, bonobo, gorilla, and orangutan using BioEdit version 7.2.0 (Hall 1999). To avoid co-amplification of *Gogo-B* alleles, we also designed a new forward primer. Encompassing complete coding region sequences, primers were manually placed in interspecies conserved regions unique to *MHC-C* with the forward primer (5′-ACTCCCATTGGGTGTCGGGTTCTAG-3′) located in the enhancer-promoter region and the reverse primer (5′-GGYGTGAAGAAATCCTGCATCTCAGTC-3′) located in the 3’UTR. Primers were designed to have melting temperatures of at least 64 °C to enhance binding specificity (Hans et al. 2017).

### Samples, LR-PCR amplifications, and sequencing

High-quality genomic DNAs extracted from 35 gorilla samples were used for the LR-PCR amplifications. These included one eastern lowland gorilla (*Gorilla beringei graueri*), one Cross River gorilla (*Gorilla gorilla diehli*), and 33 western lowland gorillas (*Gorilla gorilla gorilla*). Details of samples and DNA extractions are given in Hans et al. (2017). Two separate LR-PCR amplifications were performed on each DNA sample. In a total volume of 50 μL, LR-PCR reactions contained 1× Crimson LongAmp Taq Reaction Buffer (New England Biolabs, Frankfurt am Main, Germany), 25 μM each dNTP, 0.2 μM each forward and reverse primers, 0.1 U Crimson LongAmp Taq DNA Polymerase (New England Biolabs), and approximately 50 ng DNA template. LR-PCR amplifications started with an initial denaturation at 94 °C for 2 min followed by 40 cycles of denaturation at 94 °C for 20 s and a combined annealing and elongation step at 68 °C for 9 min and finished with an elongation phase of 15 min at 68 °C. Amplicon quality and quantity were assessed by gel electrophoresis with 10 μL of LR-PCR product analyzed in a 1.0% TAE agarose gel stained with ethidium bromide. After confirmation of successful amplification, remaining reaction volumes were individually purified using a 1.0× ratio of Agencourt AMPure XP beads (Beckman Coulter, Krefeld, Germany) following the manufacturer’s protocol and concentrations were spectrophotometrically quantified using a NanoDrop ND-1000 (Thermo Scientific, Waltham, MA, USA). Equimolar amounts of each amplicon were individually barcoded using PacBio barcoded adapters for multiplex sequencing (Pacific Biosciences, Menlo Park, CA, USA) and used to construct SMRT cell libraries according to the manufacturer’s instructions. Multiplexed libraries consisting of 35 individually barcoded amplicons were subjected to two DNA damage repair steps and exonuclease treatment. After quantification with an Agilent DNA 12000 Kit and the 2100 Bioanalyzer (Agilent Technologies, Santa Clara, CA, USA), the DNA/Polymerase Binding Kit P6 v2 (Pacific Biosciences) and the MagBead Kit v2 (Pacific Biosciences) were used to prepare libraries for sequencing on the PacBio RS II System (Pacific Biosciences). A total of two SMRT cells were sequenced, yielding 98,488 and 101,485 polymerase reads, respectively. Determination of phased consensus sequences was accomplished by the Long Amplicon Analysis (LAA) as implemented in the SMRT Analysis version 2.3.0 followed by manual trimming of primer sequences. As mentioned above, LR-PCR amplifications were performed twice for each of the 35 gorilla samples. Number of subreads per individual totaled from 829 to 2000 with an average of 1639 ± 424. Per individual and replicate, number of subreads ranged from 411 to 1000 (mean 882 ± 185). Subread coverage of consensus sequences was between 118 and 500 (mean 441 ± 106). The estimated accuracy of subreads was high with an average of 99.989% (± 0.0002, range 99.863–99.994%). Comparison of consensus sequences between replicates revealed complete identity. Novel full-length genomic coding sequences were submitted to the MHC-IPD NHP database and officially designated by the nonhuman primate MHC nomenclature committee (Maccari et al. 2017).

### Phylogenetic analyses and neutrality tests

Due to their close evolutionary history, we downloaded from the IPD-IMGT/HLA database full-length *HLA-B* and *HLA-C* coding region sequences representative of the major allelic lineages (Fukami-Kobayashi et al. 2005; Robinson et al. 2015). Full-length coding region sequences of the chimpanzee (*Patr-B* and *-C*), bonobo (*Papa-B* and *-C*), gorilla (*Gogo-B*, *-C* and *Gobe-B*, *-C*), and orangutan (*Popy-B*, *-C*, and *Poab-B*, *-C*) were retrieved from the IPD-MHC database (Maccari et al. 2017). Using the program MAFFT (Katoh et al. 2002), we generated an alignment which was used to reconstruct a neighbor-joining tree based on the Tamura-Nei model with 500 bootstrap replicates as implemented in MEGA version 6 (Tamura et al. 2013). In addition, we used the program RDP version 4 (Martin et al. 2015) for the detection and identification of recombination events in combination with domain-by-domain phylogenetic analyses to infer evolutionary relationships between *Gogo* class I genes (Hans et al. 2017). From the contig (000730_F), we extracted the extended gene sequence of the putative *Gogo-C* gene. Available genomic sequences containing the *MHC-B* or *-C* gene of human, chimpanzee, bonobo, gorilla, and orangutan were downloaded from the NCBI GenBank nucleotide database. Using rhesus macaque as outgroup, genomic sequences of *Mamu-B4*, *-B8,* and *-B9* were obtained from the macaque MHC class I region (GenBank ID: AB128049) (Kulski et al. 2004). Extended gene sequences were aligned using MAFFT and manually edited. Large insertions (≥ 100-bp) identified to be unique to a single sequence or macaque-specific were excluded (Abi-Rached et al. 2011). Following the RDP analysis, the alignment was divided into five segments, each of which was used to generate a phylogeny as described above.

We calculated homozygosity rates at individual gorilla MHC class I genes by counting the number of individuals being homozygous for a given locus divided by the total number of individuals analyzed. However, it has to be noted that gorillas have a comparatively complex MHC class I haplotype structure, in particular the *Gogo* class I *A* region exhibits haplotypic variation in gene number and content (Hans et al. 2017). As such, the gorilla MHC class I haplotype containing *Gogo-Oko* is characterized by the absence of the *Gogo-A* gene (Gleimer et al. 2011; Hans et al. 2017). Thus, given the potential hemizygous state of *Gogo-A* and *-Oko*, homozygosity rates at these loci were calculated using the number of homozygous individuals at *Gogo-A* or *-Oko* divided by the total number of individuals possessing this haplotype, respectively.

To detect departures from neutrality in the allele frequency distribution of *Gogo* class I genes, we used the program DnaSP version 5.10 (Librado and Rozas 2009) to perform the Tajima’s *D* (Tajima 1989), Fu and Li’s *D*, and Fu and Li’s *F* tests (Fu and Li 1993) on each locus separately.

## Results and discussion

Previously, we presented a detailed description of the MHC class I genes in gorillas. The combination of LR-PCR amplifications and long-read sequencing technology allowed us to characterize gorilla MHC class I genes in full-length. This approach demonstrated the ability to identify many novel gorilla MHC class I alleles as well as two previously undetected genes, thus substantially contributing to the understanding of MHC class I gene variation in this great ape species (Hans et al. 2017). Nonetheless, crucial for a comprehensive assessment of the MHC diversity is the design of primers reliably amplifying the entire allelic variation at each locus. However, in non-model organisms, such as the gorilla, extensive genomic resources or detailed information about the organization of the MHC are often not available (Babik 2010; but see Wilming et al. 2013). Although an increasing number of whole-genome studies were applied to non-model organisms, including gorillas, the nature of short-read sequencing technologies has prevented an in-depth analysis of the MHC due to its inherent complexity (Scally et al. 2012, 2013; Prado-Martinez et al. 2013; Xue et al. 2015). In contrast, the recent study by Gordon et al. (2016) utilized long-read sequencing technology to generate a high-quality assembly of the gorilla genome which substantially exceeds previously released draft genomes in accuracy and continuity. Analysis of this gorilla assembly suggests that we may have underestimated variation at *Gogo-C*, the gorilla orthologue of *HLA-C*, in our previous study (Hans et al. 2017). Thus, we here present the resequencing of *Gogo-C* aimed towards a more comprehensive description of the gorilla MHC class I gene diversity.

### Recombination has shaped the divergent Gogo-C*03:01 allele

**Fig. 1:**
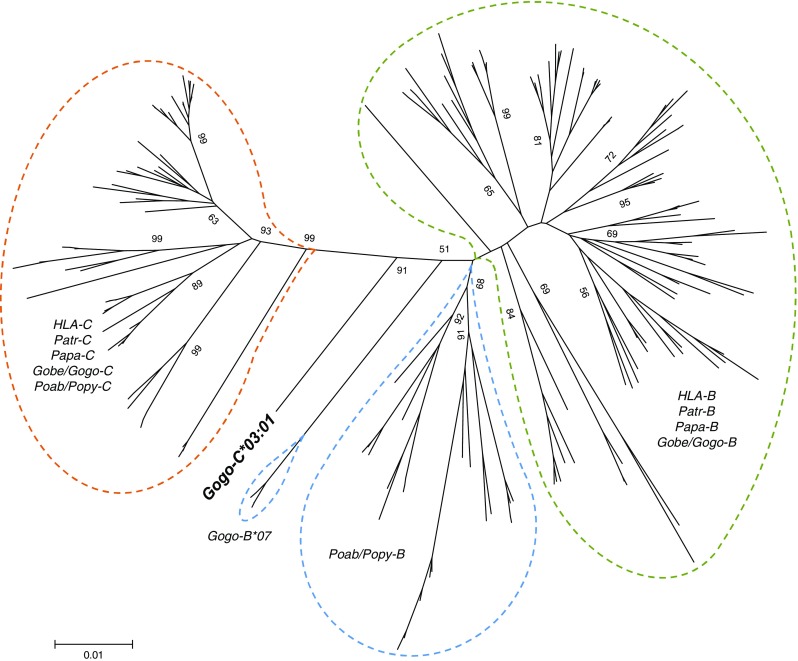
Phylogenetic tree constructed from full-length coding region sequences of *MHC-B* and *-C* genes. Relevant bootstrap values (≥ 50%) are shown. The novel allele Gogo-C*03:01 is highlighted in bold. Dashed lines in orange, green, and blue indicate the phylogenetic clusters of the orthologous *MHC-B*, *-C*, and *-B-*like genes, respectively. *HLA* human, *Patr Pan troglodytes*, *Papa Pan paniscus*, *Gogo Gorilla gorilla*, *Gobe Gorilla beringei*, *Poab Pongo abelii*, *Popy Pongo pygmaeus*

Among the 35 gorilla individuals reanalyzed, we obtained 13 genomic sequences of which 10 were completely identical to full-length *Gogo-C* alleles identified in our previous study (Hans et al. 2017). The remaining three full-length genomic sequences that have not been previously described, Gogo-C*03:01:01:01 (Genbank accession number: MF593179), -C*03:01:01:02 (MF593180), and -C*03:01:01:03 (MF593181) were identical in coding region sequences but differed at 1–2 nucleotide positions in noncoding regions. Phylogenetic analysis of full-length coding region sequences clearly segregates Gogo-C*03:01 apart from *MHC-C* alleles of humans, chimpanzees, bonobos, gorillas, and orangutans (Fig. 1). This phylogenetically divergent *Gogo-C* allele appears to be most closely related to Gogo-B*07, the additional *B*-like gene that is present on some gorilla MHC haplotypes (Hans et al. 2017).

**Fig. 2:**
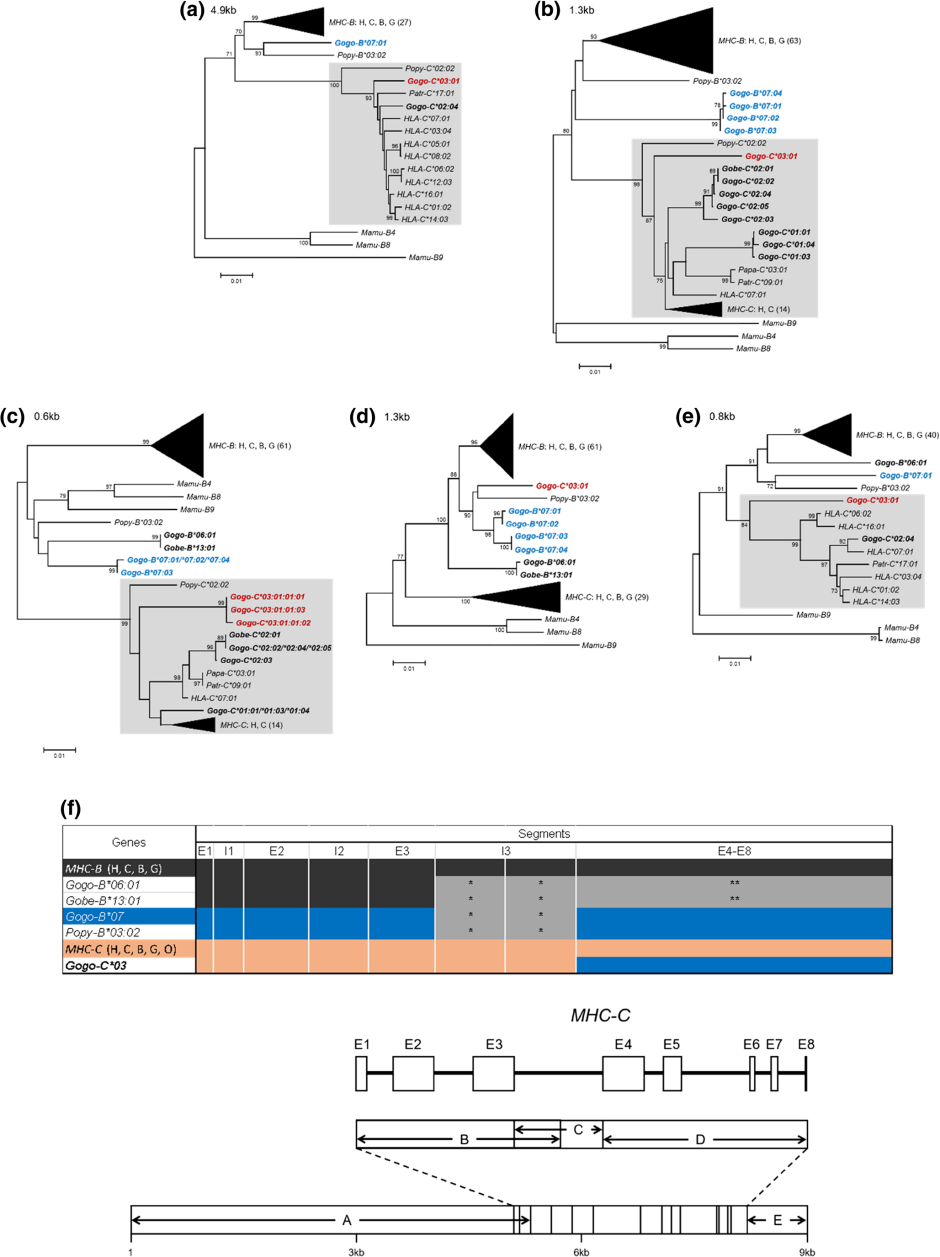
Domain-by-domain phylogenetic analysis of the newly identified Gogo-C*03:01 alleles. **a**–**e** Neighbor-joining trees constructed from five genomic segments of *MHC-B* and *-C* genes corresponding to the segments given in the schematic at the bottom. Shown in the upper left corner is the size of the genomic segment which was used for each phylogenetic reconstruction. Relevant bootstrap values (≥ 70%) are shown. Black triangles represent compressed *MHC-B* and *-C* sequences of human (H), chimpanzee (C), bonobo (B), and gorilla (G) with the total number of sequences given in parentheses. Highlighted in red are sequences of Gogo-C*03:01 alleles. Highlighted in blue are sequences of alleles of Gogo-B*07, the additional *B-*like gene present on some gorilla MHC haplotypes. Other relevant *Gogo* alleles are in bold. **f** Summary of domain-by-domain phylogenetic analyses showing the relationships between *MHC-B*, *MHC-B-*like, and *MHC-C* genes of human (H), chimpanzee (C), bonobo (B), gorilla (G), and orangutan (O). Equivalent segments between or within species have the same color. *, outgroup to both *MHC-B* and *MHC-C*, ** outgroup to *MHC-B*, *MHC-B-*like and *MHC-C*. *HLA* human, *Patr Pan troglodytes*, *Papa Pan paniscus*, *Gobe Gorilla beringei*, *Gogo Gorilla gorilla*, *Popy Pongo pygmaeus*, *Mamu Macaca mulatta*

However, intra- and interlocus recombination between members of the MHC class I multigene family can confound orthologous relationships (Jakobsen et al. 1998; Ohta 2010; Hans et al. 2017). Therefore, we investigated extended gene sequences for patterns of recombination in combination with phylogenetic analyses to infer evolutionary relationships between *Gogo* class I genes (Fig. 2). Domain-by-domain phylogenetic analysis shows that flanking segments of Gogo-C*03:01 are most closely related to *MHC-C* alleles of humans, chimpanzees, gorillas, and orangutans (Fig. 2a). This relationship extends throughout the gene (Fig. 2b–c) with the exception of the genomic segment encompassing exons 4–8 where Gogo-C*03:01 is most similar to Popy-B*03:02 and Gogo-B*07 alleles (Fig. 2d). In the genomic region downstream of the gene, Gogo-C*03:01 shares sequence similarity with *HLA-C*, *Patr-C*, and *Gogo-C* alleles (Fig. 2e). These findings demonstrate that Gogo-C*03:01 is a divergent *Gogo-C* allele which through recombination obtained segments from Gogo-B*07, the additional *Gogo-B* gene (Fig. 2f).

### Functional importance of the Gogo-C*03:01 allele

Besides antigen presentation, MHC class I molecules are involved in the regulation of NK cells via KIR. In humans, KIR recognition of HLA class I molecules is determined by amino acid motifs within the α_1_ domain. Depending on the motif, HLA class I allotypes carry either one of four mutually exclusive epitopes (A3/11, Bw4, C1, and C2) or do not permit KIR recognition (Parham et al. 2012). More specifically, as ligands for lineage II KIR, the Bw4 epitope is carried by HLA-B allotypes with arginine at position 83 while the combination of valine at position 76 and asparagine at position 80 determines the C1 epitope which is recognized by lineage III KIR. In contrast, HLA-C allotypes with asparagine or lysine at position 80 carry the C1 epitope or the C2 epitope, respectively, both of which are ligands for lineage III KIR molecules (reviewed in Parham and Moffett 2013).

**Fig. 3:**
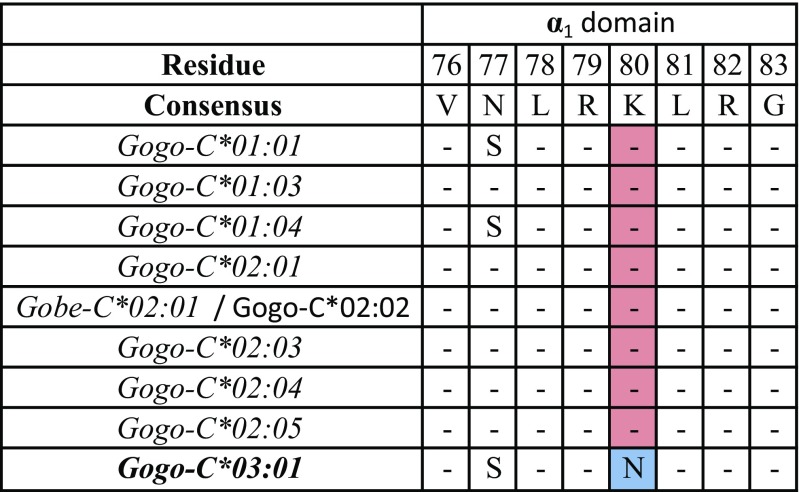
Motifs encoding KIR ligands in the sequences of Gogo-C molecules. Identity to consensus sequence is denoted by a dash. For differences from the consensus sequence, the amino acid is shown. The newly identified Gogo-C*03:01 allotype is highlighted in bold. Positions highlighted in blue and red define the C1 and the C2 epitope, respectively

It has been shown that certain combinations of MHC class I and KIR variants are associated with resistance and susceptibility to infectious diseases (Kelley and Trowsdale 2005; Parham 2005). These epistatic interactions are considered to have driven the coevolution of the MHC class I genes and their cognate KIR (reviewed in Guethlein et al. 2015). As such, the comparatively low polymorphism and cell surface expression of *HLA-C* has been attributed to its specialized function as the dominant ligand for KIR (Single et al. 2007; Older Aguilar et al. 2010). Both humans and chimpanzees harbor C1- and C2-bearing MHC-C allotypes whereas gorilla MHC-C allotypes with the C1 epitope have yet to be identified (Adams and Parham 2001; Moesta et al. 2009). Indeed, previously, we found that the C1 epitope is carried only by certain gorilla MHC-B allotypes, in particular Gogo-B*07, whereas all identified gorilla MHC-C allotypes have the C2 epitope. Here, we show that the newly identified gorilla class I allotype, Gogo-C*03:01, exclusively carries the C1 epitope at Gogo-C, as summarized in Fig. 3. We identified this allotype in 17 of the 35 gorillas analyzed, suggesting its important function in controlling NK cell responses via KIR (Table 1).

**Table 1:**
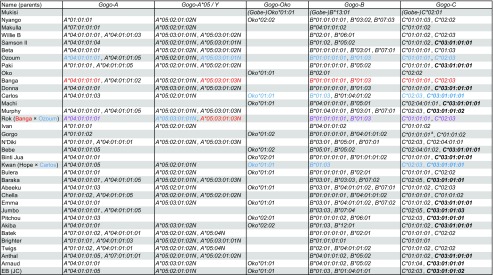
Summary of individual gorilla MHC class I genotypes identified in our present and previous study (Hans et al. 2017). Newly identified alleles from the present study are highlighted in bold. Ancestries of related individuals are shown: Alleles in red are transmitted by the mother, alleles in blue are transmitted by the father, and alleles in purple could have been transmitted by either.

^a^This allele has not been identified in our previous study (Hans et al. 2017)

Although we cannot completely rule out the possibility that we might have missed additional alleles, several lines of evidence indicate that we have identified the complete allelic diversity at *Gogo* class I genes: (i) homozygosity levels between *Gogo* class I genes are not substantially different with 18.7, 18.7, 17.1, and 14.2% of individuals homozygous at *Gogo-A*, *-Oko*, *-B,* and *-C*, respectively; (ii) many homozygous individuals have only one allele at two or more loci, presumably due to strong linkage disequilibrium between *Gogo* class I genes; and (iii) alleles identified in related individuals do not show deviations from expected inheritance patterns (Table 1). Thus, we are confident that we herewith fully complement the characterization of allelic diversity at *Gogo* class I genes.

### A selective sweep in gorillas?

**Fig. 4:**
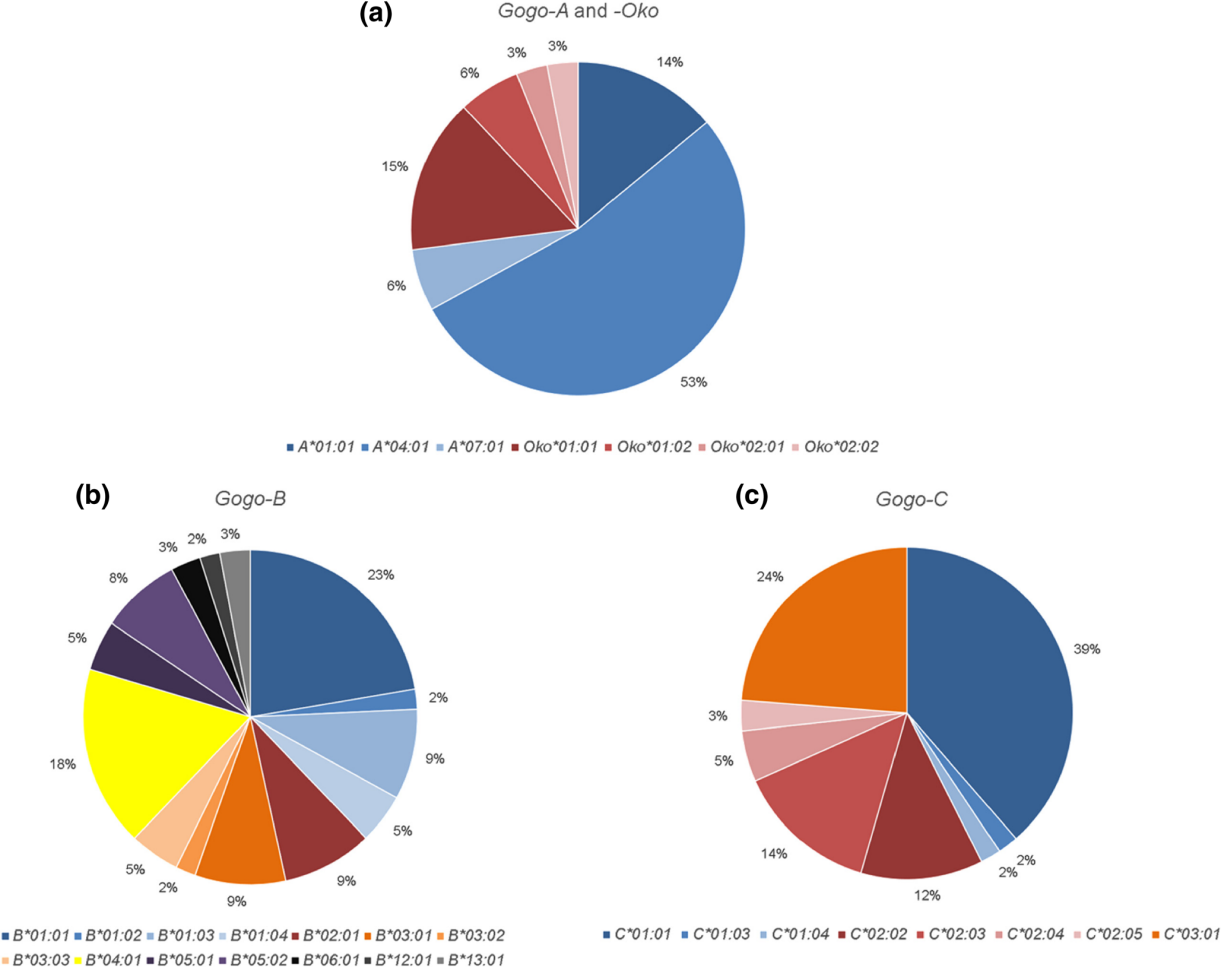
Pie charts illustrating the percentage of *Gogo* class I alleles present in the 33 unrelated gorillas analyzed. Figure shows the frequencies of **a** *Gogo-A* and *-Oko* alleles, **b** *Gogo-B* alleles, and **c** *Gogo-C* alleles

Recent genome-wide surveys of gorilla genetic variation reported a marked reduction of diversity close to the MHC suggestive of a recent selective sweep (Scally et al. 2013; Xue et al. 2015). Indeed, we have previously shown that the overall *Gogo* class I gene diversity is low relative to humans and chimpanzees despite gorillas having a comparatively complex MHC class I haplotype structure (Hans et al. 2017). Further evidence suggesting that gorillas experienced a reduction of their MHC class I repertoire comes from the uneven allele frequency distribution of *Gogo* class I genes, as illustrated in Fig. 4. Among the 33 unrelated gorillas analyzed, we show that the frequencies of the two most common *Gogo-A* alleles, Gogo-A*01:01 and -A*04:01, account for over 60% of the total variance (Fig. 4a). For the *Gogo-Oko* haplotype, the allele Gogo-Oko*01:01 has the highest proportion of the total allele frequency (15%) (Fig. 4a). The absence of a predominant allele lineage at *Gogo-B*, the gorilla orthologue of *HLA-B*, may be a result of frequent recombination promoting the exchange of sequence motifs at this locus (McAdam et al. 1994; Parham and Ohta 1996; Jakobsen et al. 1998). Indeed, phylogenetic analyses revealed the prevalence of divergent gorilla *B* locus alleles with many of them present at frequencies below or equal to 5%, suggesting the rapid diversification of *Gogo-B* alleles (Fig. 4b) (Hans et al. 2017). In contrast, the frequency spectrum at *Gogo-C* is also skewed towards high-frequency alleles: the Gogo-C*01:01 allele has a frequency of 39% whereas the novel Gogo-C*03:01 allele was identified in 16 of the 33 unrelated gorillas and, hence, accounts for 24% of the total allele frequency (Fig. 4c). However, unlike a selective sweep in which, by definition, all adaptive alleles arise from a single mutation and coalesce into a single ancestral lineage, gorillas have maintained several allelic lineages at individual *Gogo* class I genes (Wilson et al. 2014; Hans et al. 2017). In accordance, the neutrality tests applied do not indicate an excess of rare alleles characteristic of a selective sweep (Tajima 1989; Kim 2006). More specifically, with the exception of *Gogo-C*, Tajima’s *D* shows no significant deviations from neutral expectations whereas Fu and Li’s *D* and *F* statistics indicate a skew in the allele frequency distribution among *Gogo* class I genes (Table 2). Significantly positive values reflect a deficit of rare alleles which can result from balancing selection, population structure, and/or bottlenecks (Jensen et al. 2005; Biswas and Akey 2006).

**Table 2:** Summary of neutrality tests. Tajima’s *D*, Fu and Li’s *D* and F statistics were applied to *Gogo* class I genes with *, **, and *** corresponding to significance values of *p* < 0.05, *p* < 0.02, and *p* < 0.001, respectively

Locus	Tajima’s *D*	Fu and Li’s *D*	Fu and Li’s *F*
*Gogo-A*	3.53604	2.12689**	2.45849**
*Gogo-Oko*	0.93540	2.06617**	2.09826**
*Gogo-B*	0.48252	2.35116**	1.85848*
*Gogo-C*	3.53604***	2.56455**	3.53604**

Balancing selection maintains advantageous diversity in the population and is considered to be one of the main mechanisms maintaining MHC polymorphism over long evolutionary time periods, a process known as trans-species polymorphism (Bernatchez and Landry 2003; Klein et al. 2007). Polymorphism of MHC class I genes is predominantly confined to exons 2 and 3, encoding the contact residues of the antigen-binding site (ABS), typically showing elevated ratios of nonsynonymous (Ka) to synonymous (Ks) substitution rates indicative of balancing selection (Hughes and Nei 1988; Ohta 1991; Hughes and Yeager 1998; Spurgin and Richardson 2010). Previously, we analyzed Ka and Ks substitution rates within ABS of *Gogo* class I genes (Hans et al. 2017). Although significant differences between Ka and Ks were observed only for *Gogo-B*, patterns of Ka/Ks ratios suggest that balancing selection acts to maintain diversity within ABS of gorilla MHC class I genes. However, except for *Gogo-B*, absolute numbers of variable sites within MHC class I alleles are substantially lower in gorillas than in chimpanzees and humans. Indeed, as reflected by an interspecies comparison, there is only limited allelic variation within lineages of gorilla MHC class I genes including *Gogo-B* (Hans et al. 2017). Similarly, differences between newly identified Gogo-C*03:01 alleles are only due to single nucleotide substitutions in noncoding regions. Furthermore, many allelic lineages that are present in chimpanzees and/or humans are absent in gorillas, contrary to expectations given the trans-species mode of evolution (Trowsdale 1995; Klein et al. 2007; Hans et al. 2017). Thus, although balancing selection may be acting on short evolutionary time scales, the overall patterns observed at *Gogo* class I genes are also suggestive of ancient and/or recent demographic changes in gorillas.

Although previous studies revealed substructure within gorilla populations, particularly within western lowland gorillas, these findings are unlikely to explain the high homogeneity of *Gogo* class I alleles identified in our samples (Clifford et al. 2004; Thalmann et al. 2007; Anthony et al. 2007; Scally et al. 2013; Fünfstück et al. 2014). However, it has to be noted that we cannot completely exclude the possibility of sampling from genetically homogeneous populations, despite the knowledge of geographical origins of most gorillas (Hans et al. 2017). Nevertheless, it has been suggested that the present-day substructure observed within western lowland gorillas rather reflects ancestral population structure (Thalmann et al. 2007). Postglacial forest recovery during the Pleistocene allowed the expansion and admixture between previously separated populations, thereby promoting genetic diversity (Anthony et al. 2007; Thalmann et al. 2007; Scally et al. 2013).

Indeed, although western lowland gorillas show the highest levels of genetic diversity relative to other great ape species, there are indications of a severe genetic bottleneck (Prado-Martinez et al. 2013). Recent demographic inferences revealed that western lowland gorillas experienced a fourfold decrease in their effective population size (*N*_*e*_) approximately 30,000–50,000 years ago (Prado-Martinez et al. 2013; McManus et al. 2015; Gordon et al. 2016). In accordance, distributions of genomic runs of homozygosity (ROH) in gorillas are indicative of a history of inbreeding with patterns similar to those in human populations that have undergone strong genetic bottlenecks (Kirin et al. 2010; Prado-Martinez et al. 2013; Xue et al. 2015). Furthermore, consistent with our observations at *Gogo* class I genes, genome-wide analysis of the allele frequency spectrum revealed a deficit of rare alleles, further indicating that western lowland gorillas experienced a recent episode of population contraction (Scally et al. 2013). In accordance, alleles at *Gogo* class I genes accumulated only limited polymorphisms within both coding and noncoding regions (Hans et al. 2017). Indeed, previous estimates inferred that the western lowland gorilla population has decreased by more than 60% in the past 20–25 years (Vogel 2007). Besides habitat fragmentation and degradation, major threats responsible for the drastic decline of gorilla populations include poaching and infectious diseases, in particular Ebola (Walsh et al. 2003; Junker et al. 2012). In some regions, the Ebola epidemic has killed more than 90% of the western lowland gorillas, which has prompted their conservation status change to “critically endangered” (Bermejo et al. 2006; Hopkin 2007; Le Gouar et al. 2009). Even more dramatic, recent census estimates predict a decline of western gorillas by over 80% in the next three generations, a scenario which would bring gorillas to the brink of extinction (Maisels et al. 2016).

Taken together, these findings strongly suggest that the low variation observed at *Gogo* class I genes resulted from successive genetic bottlenecks that caused the loss of MHC diversity in gorillas. Following this scenario, we propose that after the initial reduction of the *Gogo* class I repertoire, functionally divergent alleles were maintained in the population through balancing selection, as can be seen from the comparatively high nucleotide diversity values (Hans et al. 2017). Indeed, similar patterns have been observed in other species that have undergone severe bottlenecks (Hedrick et al. 2000a, 2000b; Sommer 2005; Ellison et al. 2012). Subsequently, however, *Gogo* class I diversity became further decreased during the recent bottleneck(s), as reflected by the low polymorphisms that accumulated within allelic lineages of *Gogo* class I genes.

In sum, we demonstrated the advantage of high-quality genomes for a comprehensive description of the MHC diversity in non-model organisms, such as the gorilla. Reassessment of the *Gogo-C* diversity showed that gorillas, unlike previous suggestions, exhibit a high-frequency allele exclusively encoding a Gogo-C allotype with the C1 epitope. Furthermore, we propose that the low diversity at *Gogo* class I genes can be best explained by drastic demographic changes gorillas experienced in the ancient and recent past. However, we strongly encourage future studies to incorporate more sensitive haplotype-based analyses to test this hypothesis.

## Electronic supplementary material

ESM 1 (PDF 133 kb)
