# Supplementary material for: Discovery of gorilla MHC-C expressing C1 ligand for KIR
Source: Immunogenetics. 2017 Nov 3;70(5):293–304. doi: 10.1007/s00251-017-1038-y (PMC5899755; doi:10.1007/s00251-017-1038-y)
Supplement: Supplementary file 1 — (PDF 133 kb) [file 251_2017_1038_MOESM1_ESM.pdf]

Electronic Supplementary Material 1. Illustration of primer-template misalignment. Virtual genotyping with the *Gogo-C* primers used in our previous study revealed that the contig sequence contains a single base pair insertion (highlighted in red) causing mismatches at the 3'-end of the reverse primer (Hans et al. 2017).

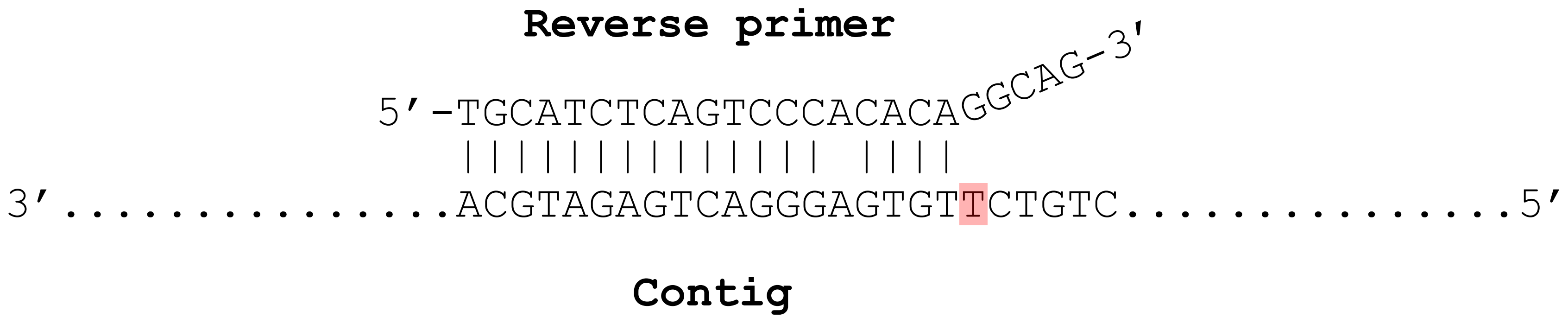

Reference

Hans JB, Bergl RA, Vigilant L (2017) Gorilla MHC class I gene and sequence variation in a comparative context. Immunogenetics. doi: 10.1007/s00251-017-0974-x
